# Modulation of Igβ is essential for the B cell selection in germinal center

**DOI:** 10.1038/srep10303

**Published:** 2015-05-18

**Authors:** Kagefumi Todo, Orie Koga, Miwako Nishikawa, Masaki Hikida

**Affiliations:** 1 Center for Innovation in Immunoregulative Technology and Therapeutics, Graduate School of Medicine, Kyoto University Yoshidakonoecho, Sakyoku, Kyoto 606-8501, Japan

## Abstract

The positive and negative selection of antigen-reactive B cells take place in the germinal center (GC) during an immune responses. However, the precise molecular mechanisms underlying these selection machineries, including the involvement of antigen receptor signaling molecules, remain to be elucidated. We found that expression levels of Igα and Igβ, which are the essential components of B cell antigen-receptor complex, were differentially regulated in GC B cells and that the expression of Igβ was more prominently down-regulated in a portion of GC B cells. The suppression of Igβ down-regulation reduced the number of GL7^+^GC B cells and the affinity maturation in T-dependent responses was markedly impaired. In addition, the disease phenotypes in autoimmune-prone mice were ameliorated by blocking of Igβ down-regulation. These results suggest that Igβ down-regulation is involved in the normal positive selection in GC and the accumulation of autoreactive B cells in autoimmune-prone mice.

The B cell antigen receptor (BCR) is a protein complex that consists of a membrane-bound immunoglobulin (Ig) molecule and the signal transducer, an Igα/Igβ hetero-dimer, and other signaling molecules^1^,^2^,^3^. It is well known that the Igα/Igβ hetero-dimer is required for the expression of membrane-bound Igμ chains on the surface of preB cells^1^,^4^,^5^. Furthermore, Igμ chains expressed on the cell surface of B lineage cells in association with Igα/Igβ hetero-dimer play essential roles in both differentiation and survival of preB cells and mature B cells^6^,^7^. In addition, it has been shown that cell surface expression of the Igα/Igβ hetero-dimer not only supports the expression of cell surface Igμ chains, but also the signal through this complex is further required for the differentiation and survival of B lineage cells^8^,^9^,^10^. Hence, it has been widely believed that both Igα and Igβ are expressed in B lineage cells during all maturation stages.

After completion of differentiation, mature B cells participate in the humoral immune responses. One of the hallmarks of the humoral immune response is the formation of germinal centers (GCs) following the activation of B cells by an antigen under the influence of T cells^11^,^12^,^13^. It is widely known that GC B cells can be classified into two compartments namely centroblasts and centrocytes. Centroblasts are observed in the dark zone and they lack or express only low levels of surface Ig. These cells proceed with somatic hypermutation of their antibody variable genes and proliferate rapidly, which contribute to the clonal expansion. In contrast, centrocytes are relatively small non-dividing cells with surface Ig in the light zone where positive and negative selection take place^14^. A combination of somatic hypermutaion, clonal expansion, and selection leads a part of GC B cells to acquire a BCR with higher affinity for the antigen, which results in the affinity maturation of serum antibodies. It has been widely shown that a portion of GC B cells, mainly consisting of centroblasts, reduces their surface BCR expression during these processes. Thus, it can easily be predicted that BCR-associating molecules, including Igα and Igβ, are down-regulated in these cells. Indeed, it has been reported that expression of both Igα and Igβ was down-regulated in the germinal center (GC) B cells^15^,^16^,^17^. However, it has not been determined whether the modulation of these signaling molecules has as-yet-unknown physiological roles or simply reflects BCR down-regulation.

In this study, we demonstrated that expression levels of Igα and Igβ, were differentially regulated in GC B cells and that the expression of Igβ was more prominently down-regulated in a part of GC B cells. Furthermore, this down-regulation of Igβ is involved both in the effective positive selection in GC B cells and the accumulation of autoreactive B cells in autoimmune-prone mice.

## Results

### The expression of Igβ is down-regulated in GC B cells

**Figure 1:**
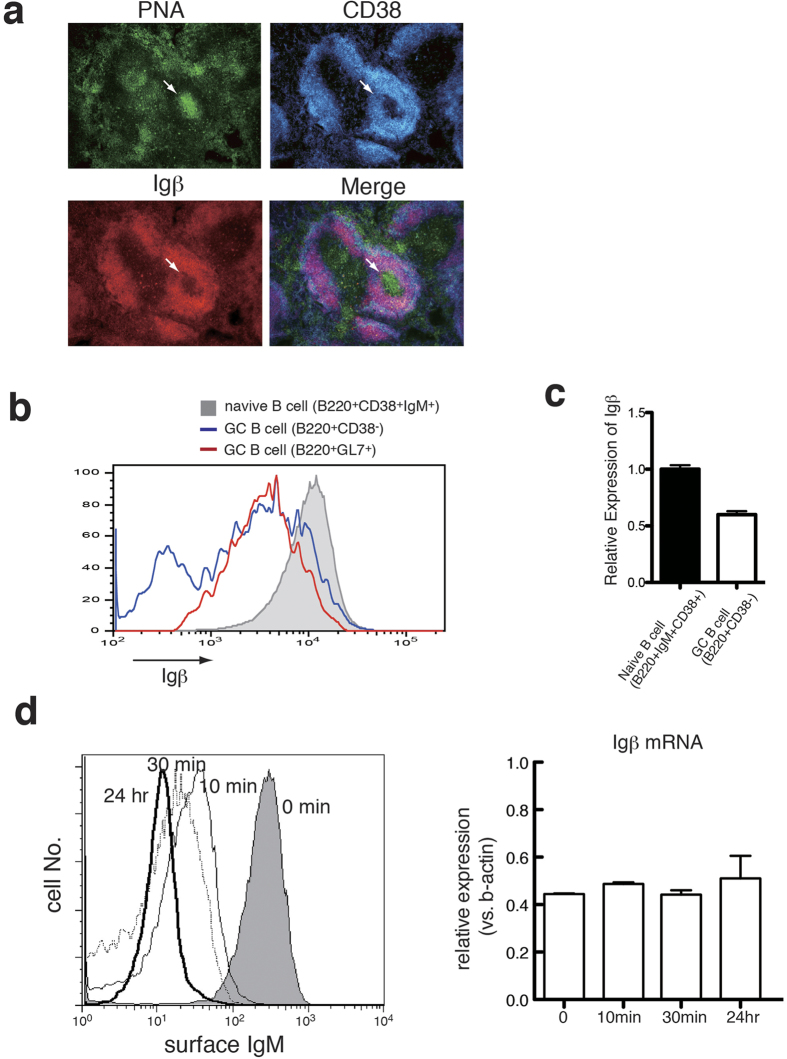
Down-regulated Igβ expression in the germinal center. (**a**) Frozen sections of the spleen from B6 mice, 10 days after the immunization with NP-CGG precipitated in alum, were stained with PNA (green) and antibodies to Igβ (red) and CD38 (blue). (**b**) Naïve B cells (shaded), B220^+^CD38^−^ cells (blue line) and B220^+^GL7^+^cells (red line) were analyzed for the expression level of Igβ. (**c**) Quantitative RT-PCR of Igβ mRNA in sorted naïve B cells (B220^+^IgM^+^CD38^+^) and GC B cells (B220^+^CD38^−^) from B6 mice 10 days after the immunization with NP-CGG. Cells were stained with propidium iodide to exclude dead cells and apoptotic cells. Data were normalized to the expression levels of the β-actin transcript. (**d**) Surface IgM in mouse splenic B cells was cross-linked with 10 ug/ml anti-IgM F(ab’)_2_ to induce endocytosis for indicated periods of time and remaining levels of cell surface IgM were analyzed (**left**). Expression levels of Igβ mRNA were analyzed by RT-qPCR in B cells whose surface IgM was down-modulated by cross-linking for the indicated periods (**right**).

It has been reported that Igβ is ubiquitously expressed in both immature and mature B cells. However, it has not been fully investigated whether Igβ is also expressed constantly in B cells during immune responses, such as in GC B cells. To clarify this point, we initially analyzed the expression of Igβ in the spleen from immunized mice by immunohistochemical staining. Ten days post immunization with NP-CGG, PNA^+^CD38^−^ GCs were clearly detected (Fig. 1a). When compared with the follicular B cells, B cells in GCs were only weakly stained by anti-Igβ antibodies (Fig. 1a). Spleen cells from immunized mice were further analyzed by flow cytometer to confirm the down-regulation of cell surface Igβ. As shown in Fig. 1b, naïve B cells (B220^+^CD38^+^IgM^+^) expressed Igβ at high levels (MFI = 9.7 × 10^3^), as expected. In contrast, the levels of Igβ decreased in GC B cells identified with either surface markers CD38 or GL7 (B220^+^CD38^−^: MFI = 2.1 × 10^3^ and B220^+^GL7^+^: MFI = 3.5 × 10^3^). Quantitative RT-PCR revealed that the level of Igβ mRNA in non-apoptotic GC B cells was reduced by approximately 50% of that in naïve B cells (Fig. 1c), suggesting that the cell surface expression of Igβ is controlled at the transcription level to some extent. According to a previous report^15^ and the present data (see below), the expression of Igβ in GC B cells clearly correlated with the down-modulation of IgM. Thus, to clarify whether this down-regulation of Igβ in GC B cells simply reflects the decrease of surface BCR expression, we examined the levels of Igβ mRNA following the endocytosis of BCR *in vitro* (Fig. 1d). In comparison with the decreased surface IgM levels after BCR cross-linking, expression of Igβ mRNA was sustained at a constant level. Although cell surface level of Igβ correlates well with the endocytosis of IgM, these results suggest that there is an additional regulatory mechanism for the down-regulation of Igβ mRNA *in vivo* independent from BCR endocytosis induced by simple cross-linking (see below).

### GC B cells can be classified into several stages based on their surface Igβ expression

**Figure 2:**
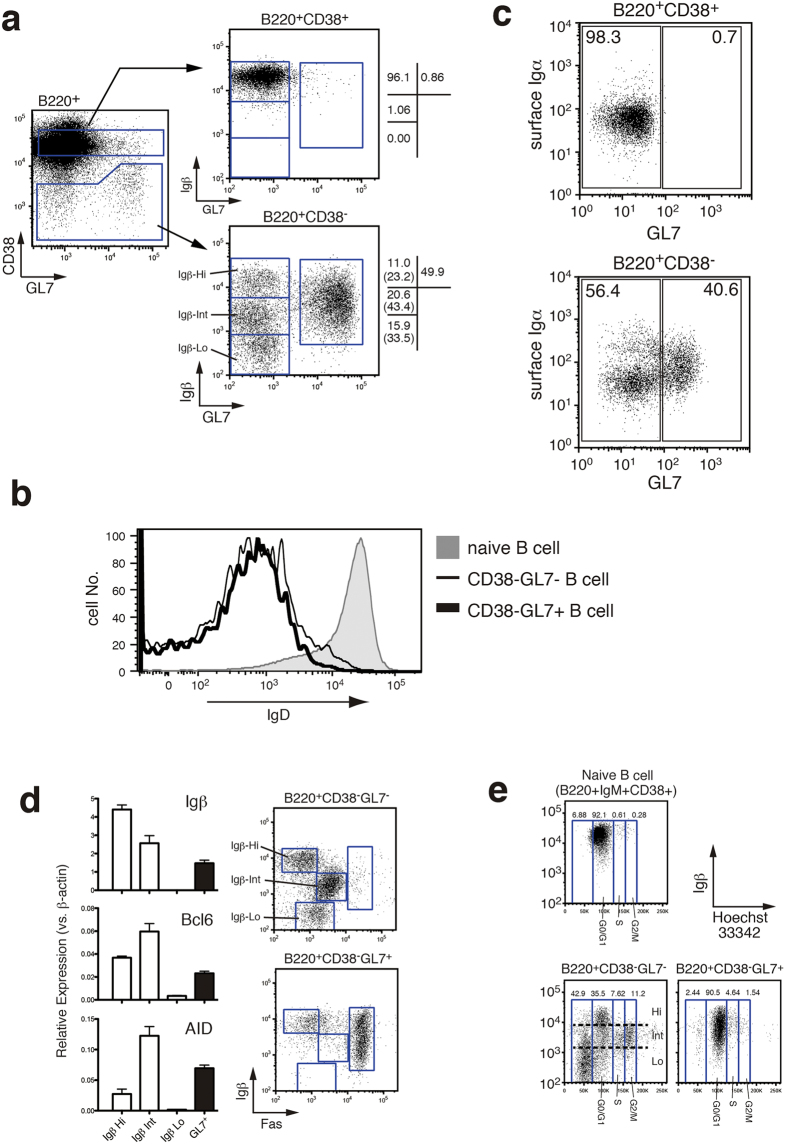
Classification of GC B cells by expression level of Igβ. (**a**) Flow cytometric analysis of splenocytes from immunized B6 mice. B220^+^B cells (left) cells were further gated to B220^+^CD38^+^cells (**top right**) and B220^+^CD38^−^ cells (**bottom right**) and analyzed for the expression levels of Igβ and GL7. The numbers indicate the percentage of cells in each square, and the number in the parenthesis indicate the percentage of cells in each square within the CD38^−^GL7^−^ cells. (**b**) Suppressed IgD expression in B220^+^CD38^−^GL7^−^ and B220^+^CD38^−^GL7^+^B cells. (**c**) Expression of cell surface Igα in GC B cells. Cell surface expression levels of Igα and GL7 were examined in B220^+^CD38^+^naive B cells (**top**) and B220^+^CD38^−^ GC B cells (**bottom**) by staining with anti-GL7 mAb in combination with rabbit polyclonal antibody raised against extracellular portion of Igα. Numbers indicate the percentages of the cells within the gates. (**d)** Transcription levels of Igβ, Bcl6 and AID in GC subsets (**left**) and surface Fas expression in B220^+^CD38^−^GL7^−^ and B220^+^CD38^−^GL7^+^GC subsets (**right**). RT-qPCR were carried out in sorted B220^+^CD38^−^GL7^−^IgβHi cells (Igβ-Hi), B220^+^CD38^−^GL7^−^IgβInt cells (Igβ-Int), B220^+^CD38^−^GL7^−^IgβLo cells (Igβ-Lo) and B220^+^CD38^−^GL7^+^cells (GL7^+^) 10 days after the immunization. (**e**) Cell cycle analysis of GC B cells. Splenocytes from immunized B6 mice were stained with Hoechst 33342 dye in combination with antibodies to Igβ and indicated surface markers, followed by FACS analysis. Naive B cells (**top**), B220^+^CD38^−^GL7^−^ (**bottom left**) and B220^+^CD38^−^GL7^+^B cells (**bottom right**) were analyzed.

When B220^+^CD38^−^ B cells from immunized mice were analyzed for GL7 expression, the resulting population could be classified into GL7^+^ and GL7^−^ subsets (Fig. 2a, left). Both of these subsets were IgD^−^, another GC B cell marker, suggesting that these subsets are both GC B cells (Fig. 2b). The B220^+^CD38^−^GL7^+^B cells formed a unique subset whose expression level of Igβ was 1/4 to 1/5 of that in B220^+^CD38^+^ naive B cells (Fig. 2a, right bottom). In contrast, CD38^−^GL7^−^ B cells could be further divided into three subpopulations based on their surface level of Igβ. Approximately 23% of the CD38^−^GL7^−^ cells expressed Igβ at a level similar to that in naive B cells (Igβ-Hi cells), 43% of CD38^−^GL7^−^ B cells expressed Igβ at a level of 1/8 to 1/10 of the level in naïve B cells (Igβ-Int cells), and 34% of these cells did not express or, at most, expressed low levels of Igβ (Igβ-Lo cells) (Fig. 2a, right bottom). In contrast, the cell surface expression of Igα was only slightly down-regulated in the CD38^−^GL7^−^ B cell subset (MFI = 42.2) as compared with naive B cells (MFI = 55.7), and the level of Igα was not down-regulated in CD38^−^GL7^+^B cells (MFI = 65.8) (Fig. 2c). These results are consistent with the previous histological analysis of human lymph nodes reported by others^17^, strongly suggesting that expression of Igα and Igβ are differentially regulated in GC B cells.

To further confirm that this B220^+^CD38^−^GL7^−^ population represents the subsets of GC B cells, we analyzed the expression of Bcl6 and AID (Fig. 2d, left). The expression of Igβ mRNA correlated well with the cell surface Igβ level, which again suggests that Igβ expression in GC B cells is regulated primarily at the transcription level. Bcl6 and AID were highly expressed in the Igβ-Hi, Igβ-Int and GL7^+^ cells. These results clearly demonstrate that the Igβ-Hi and Igβ-Int B cell subsets were indeed GC B cells. We also examined the expression level of Fas, which has been reported to be expressed in activated and GC B cells^18^,^19^ (Fig. 2d, right). High levels of Fas expression were detected in a majority of the GL7^+^B cells. Among the GL7^−^ subpopulation, the Igβ-Int population significantly up-regulated Fas expression suggesting that they are either a part of the GC B cells or at least of the activated B cells. We also checked the expression level of PD-1 in these cells and found that these Igβ-Int cells expressed only quite a low level of PD-1 (Fig. S1a), suggesting that Igβ-Int cells are a distinct population from the reported memory B cell fraction^20^. In addition, this B220^+^CD38^−^GL7^−^ population was confirmed to be CD138^−^ indicating that they were not plasma cell committed cells (data not shown). We further examined the expression levels of several known markers for GC B cells and memory B cells in B220^+^CD38^−^GL7^−^ B cells and found that Igβ-Hi cells do not express memory surface markers (Fig. S1b)

To identify the proliferating cells among the aforementioned GC subpopulations, spleen cells from immunized mice were stained with the membrane-permeable DNA staining dye Hoechst33342 (Fig. 2e). Most IgM^+^CD38^+^B cells (>90%) were in the G0/G1 phase and a small numbers of cell were in the S and G2/M phase (Fig. 2e, top). In contrast, a significant number of the GL7^+^B cells (6%-7%) were in the S + G2/M phase (Fig. 2e, bottom right). On the other hand, most of the cells in the S + G2/M phase were Igβ-Int cells belonging to the B220^+^CD38^−^GL7^−^ population. Considering that GC B cells consisted of similar number of GL7^+^and GL7^−^ populations (Fig. 2a, right bottom), these results may suggest that the major proliferating cells in the GC belong to the GL7^−^Igβ-Int B cell population (Fig. 2e, bottom left). Furthermore, it was shown that most of the Igβ-Lo cells belonged to the sub-G0 phase indicating that these cells are apoptotic (Fig. 2e, bottom left).

**Figure 3:**
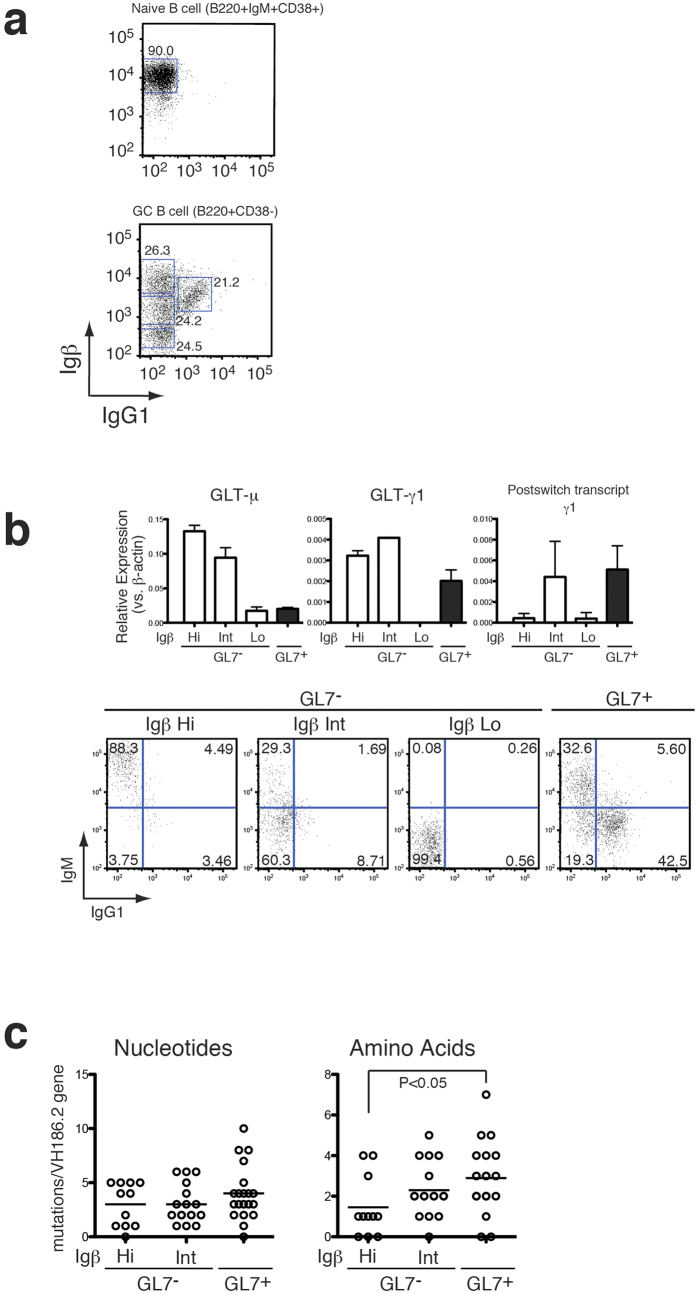
Class switching events in GC B cells **(a)** Expression of IgG1 and Igβ in B220^+^IgM^+^CD38^+^naive B cells (**top**) and B220^+^CD38^−^ GC B cells (**bottom**). (**b**) Expression levels of germline transcript and surface expression of IgM and IgG1 in the GC B cell subsets. Expression of IgM germline transcript (GLT-μ), IgG1 germline transcript (GLT-γ1) and IgG1 postswitch transcript were assessed by RT-qPCR in each population, respectively (**top**). Cell surface expression of IgM and IgG1 in each GC subset was examined (**bottom**). (**c**) Frequency of somatic hypermutation in VH186.2 gene of GC B cell subsets. Number of nucleic acid mutations (**left**) and amino acid mutations (**right**) are shown.

We next examined the expression of surface Igβ in combination with surface IgG1 instead of GL7. The level of Igβ changed dramatically along with the acquisition of surface IgG1 in the B220^+^CD38^−^ B cells, which prompted the need to analyze class switching in each population in detail (Fig. 3a, bottom). Thus, the expression of immunoglobulin mRNA was tested to clarify the class switching status of both GL7^−^ and GL7^+^ subsets identified in Fig. 2a. As the result, high levels of Iμ germline transcripts were detected in the GL7^−^ Igβ-Hi and Igβ-Int cells. Since it has been reported that Iμ germline transcripts were constitutively transcribed from the IgM locus in the germline configuration^21^ (Fig. 3b, top), it is apparent that these populations include IgM-bearing non class-switched B cells as a major population. Comparable levels of Iγ1 germline transcripts and γ1 post-switch transcripts were detected in Igβ-Int cells when compared with GL7^+^ cells (Fig. 3b, top). Since it has been reported that expression of germline transcripts take place prior to DNA rearrangement^22^, these results suggest that class switching from IgM to IgG1 may take place in both the GL7^−^Igβ-Int population and the GL7^+^ stage. In accordance with these data, nearly 90% of the cells in the GL7^−^Igβ-Hi population expressed IgM on their surface, including the population begining to down-regulate IgM (Fig. 3b, bottom left). In contrast, around 60% of the Igβ-Int population expressed only a low level of IgM and a portion of these cells (8.7%) begin to express a low, although significant, level of IgG1 on their surface and IgG1 single-positive cells were clearly detected in the GL7^+^ population (Fig. 3b, bottom right). According to a previous report^23^, the stimulation of IgM^+^B cells with anti-CD40 or CD40 ligand together with IL-4 induced only GL7^+^B cells concomitantly with class switching to IgG1. A straightforward interpretation for the previous and the aforementioned results is that, in addition to the population that became IgM^−^GL7^+^ class-switched cells following the IgM^+^GL7^+^ population, a significant portion of IgM^+^GL7^−^ GC B cells might differentiate to IgM^−^GL7^+^ class-switched cells via the IgM^dull^GL7^−^Igβ-Int stage. Indeed, both IgM^+^GL7^+^ and IgM^dull^GL7^−^ GC B cell subsets were detected (Fig. S2). This result may suggest the existence of a novel class-switching pathway distinct from *in vitro* class-switching where B cells differentiate from IgM^+^GL7^−^ to IgM^dull^GL7^+^ switched B cells through IgM^+^GL7^+^B cells. In the *in vitro* system, it was shown that the considerable number of somatic hypermutations in the Ig gene were detected only when AID was over expressed^23^. This is consistent with our results that Igβ-Int cells expressed higher level of AID (Fig. 2d).

To approach the speculation that Igβ-Int GL7^−^ B cells may contribute to positive selection in the GC, the frequency of somatic hypermutation in each subset was analyzed (Fig. 3c). On day 10 post-immunization of NP-CGG, nucleotide mutations in VH186.2 were readily detected in Igβ-Hi cells and the frequency slightly increased in the Igβ-Int and GL7^+^ cells, although the increase was not statistically significant (Fig. 3c, left). In parallel, amino acid replacements were significantly accumulated in the GL7^+^ cells as compared with Igβ-Hi cells. These results suggest that somatic hypermutations accumulate throughout the CD38^−^ stage, including both the GL7^−^ and GL7^+^ populations, and that affinity maturation may proceed in the Igβ-Int cells (Fig. 3c, right).

### Blocking of Igβ down-regulation in GC B cells impairs GC formation, efficient Ig production and affinity maturation

To further evaluate the physiological roles of Igβ down-regulation, B cell-specific Igβ-transgenic (Igβ-Tg) mice were generated (Fig. S3). Between the established two Tg lines, the line with higher expression of exogenous Igβ (line #1) was primarily analyzed. Over all differentiation of B cells in these mice was normal, except an increase in B220^dull^CD43^−^ pre-B cells was observed (Fig. S3d). Furthermore, there were no significant differences in spontaneous Ig titers between transgenic mice and wild-type littermate controls (Fig. S3e). In addition, endocytosis of IgM complex by cross-linking of BCR was not affected in these transgenic mice (data not shown).

**Figure 4:**
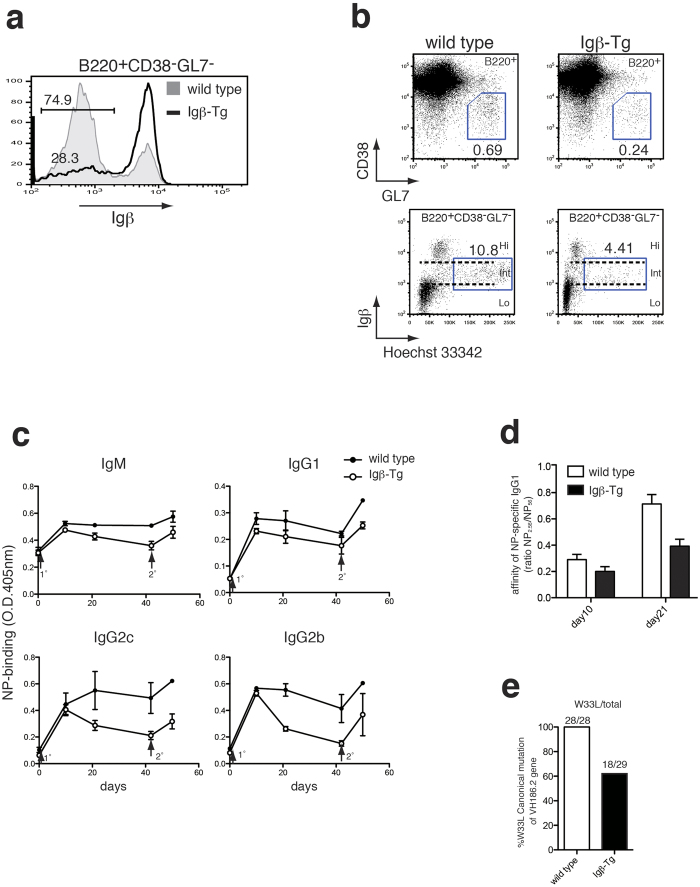
Forced expression of Igβ in B cells impaired the antibody production and affinity maturation. (**a**) Expression of Igβ in B220^+^CD38^−^GL7^−^ splenocytes from immunized Igβ-Tg (Igβ-Tg; line) and wild-type litter mates (wild type; shaded). (**b**) Expression of CD38 and GL7 in B220^+^spleen cells from immunized wild type litter mates (**top left**) and Igβ-Tg mice (**top right**). Cell cycle analysis of CD38^−^GL7^−^ GC B cells from wild type litter mates (**bottom left**) and Igβ-Tg mice (**bottom right**). Numbers indicate the percentages of cells in each gate. (**c**) Serum anti-NP titers in each isotype after the immunization. Igβ-Tg (open circle) and wild type litter mates (closed circle) were immunized with NP-CGG on day 0 (1°) and boosted 42 d later (2°). Sera were collected at indicated day and assayed by ELISA as described in Methods section. Error bars indicate SEM for each group. Each group consists of 5 mice. Representative data from 3 repeated experiments is shown. (**d**) Affinity maturation of anti-NP IgG1 antibody in NP-CGG immunized Igβ-Tg (closed bar) and wild type litter mates (open bar). Error bars indicate SEM for each group. (**e**) Decreased frequency of somatic hypermutation in immunized Igβ-Tg mice. CD38^−^GL7^+^GC B cells were isolated from Igβ-Tg and wild type litter mates 21 days after the immunization. Total RNA was extracted and reverse transcribed to obtain cDNA samples. VH186.2 gene was then amplified and sequenced for the mutation analysis.

Frequency of Igβ down-regulated cells in the B220^+^CD38^−^GL7^−^ B cells from the immunized Igβ-Tg mice (28.3%) decreased significantly compared to that in the wild-type littermate controls (74.9%), which is consistent with the results obtained in C57BL/6 mice (Figs. 4a and 2a). This finding indicates that the transgene was expressed in the expected manner, allowing us to analyze the function of Igβ down-regulation in GC B cells.

Next, the frequency of the GC B cell subsets defined above was enumerated. Normal GC formation was observed by histochemical analysis (Fig. S3f). The frequency of B220^+^CD38^−^GL7^+^ cells in the immunized spleen of the Igβ-Tg mice decreased to approximately 56% of that of wild-type mice (0.69% to 0.24%) (Fig. 4b top). In addition, the cell cycle status of the B220^+^CD38^−^GL7^−^ B cells was examined and the proportion of proliferating cells was reduced from 10.8% to 4.4% (Fig. 4b, bottom).

These results suggest the involvement of Igβ down-regulation in the differentiation of GC B cells to proliferating cells, which may affect clonal expansion and subsequent affinity maturation. To investigate this possibility, NP-specific Ig titers and their relative affinity in Igβ-Tg mice following immunization with NP-CGG were examined. As the result, there were modest reductions in the NP-specific IgM and IgG1 levels and the impairment was more prominent in the IgG2b and IgG2c levels in Igβ-Tg mice after both primary and secondary immunizations (Fig. 4c). On day 21 post-immunization, the relative affinity of IgG1 for NP hapten was significantly reduced in Igβ-Tg mice (Fig. 4d). Consistent with these data, the frequency of W33L replacement, which reflects the affinity maturation of the VH186.2 variable region to the NP hapten, among the over all mutations in the VH186.2 gene was reduced in Igβ-Tg mice (Fig. 4e).

### Igβ down-regulation in GC B cells is partly mediated by IL-21

**Figure 5:**
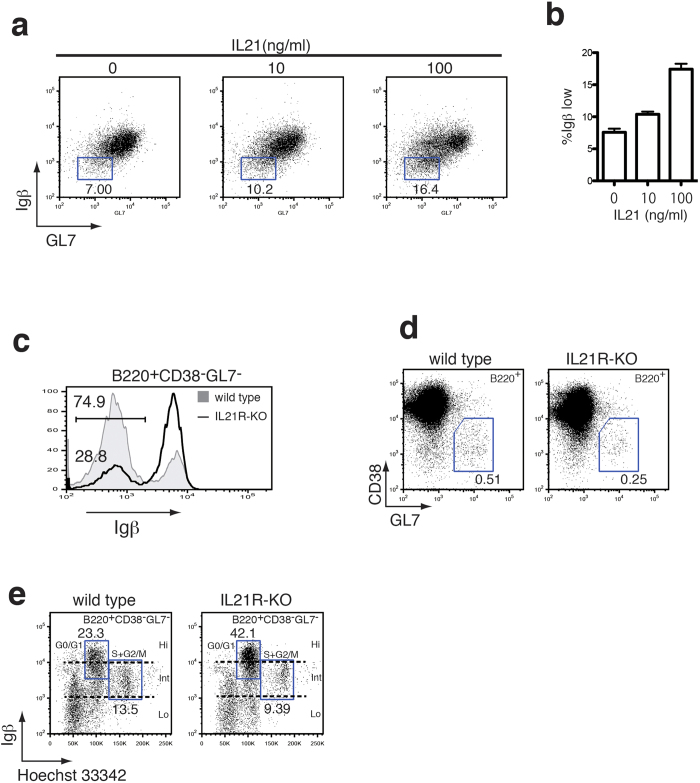
Impaired GC formation by inhibition of IL-21-mediated Igβ down-regulation. (**a**) Expression of Igβ in activated splenic B cells supplemented with IL-21. Splenocytes depleted of T cells were cultured with IL4 (10 ng/ml), and anti-CD40 antibody (1 μg/ml) supplemented with indicated concentrations of IL-21 for 6 days. Expression levels of Igβ and GL7 were analyzed by flow cytomeric analysis. The numbers under the square gate indicate the percentages of Igβ down-regulated population in the gate. Representative data from 3 experiments is shown. (**b**) Average percentages and SEM of Igβ low cells from repeated experiments as in (**a**) were shown. (**c**) Flow cytomtric analysis of Igβ expression in B220^+^CD38^−^GL7^−^ splenocytes from immunized wild type (shaded) and IL-21R-deficient (line) mice. (**d**) Flow cytometric analysis of the splenic B cells (B220^+^) from wild type litter mates (**left**) and IL-21R-deficient mice (**right**) 10 days after the immunization with NP-CGG. The numbers indicate the percentage of CD38^−^GL7^+^ cells within the gate. (**e**) Cell cycle analysis of B220^+^CD38^−^GL7^−^ GC B cells from immunized IL-21R-deficient mice. Splenocytes from wild type litter mates (**left**) and IL21R-deficient mice (**right**) 10 days after the immunization were stained with Hoechst 33342 dye in combination with antibodies to Igβ.

It has recently been reported that the absence of IL-21 signaling profoundly affects the T-dependent B cell response through the impairment of the GC reaction including antigen-specific IgG titer and affinity maturation^24^,^25^,^26^. To examine the possibility that IL-21 may exert its function by modulating the Igβ level in GC B cells, it was first evaluated whether IL-21 has the potential to induce Igβ down-regulation in purified B cells. Activation by IL-4 and an anti-CD40 antibody for 6 days, with or without IL-21, revealed that the frequency of Igβ down-regulated cells increased in an IL-21 dose-dependent manner (Fig. 5a,b). When cell surface expression of CD138 was analyzed in these cells, up-regulation of CD138 was not detected in these Igβ down-regulated B cells (Fig. S1c, right). These results indicate that IL-21 has the potential to directly act on activated B cells to regulate Igβ expression.

To further examine the involvement of IL-21 in the down-regulation of Igβ *in vivo*, the expression level of Igβ in GC B cells from IL-21 receptor-deficient (IL-21R-KO) mice^24^ was analyzed. As shown in Fig. 5c, the down-regulation of Igβ was significantly impaired in the immunized IL-21R-KO mice. Furthermore, the frequency of CD38^−^GL7^+^B cells in the spleen from IL-21R-KO mice was reduced to approximately 50% as compared with that of wild type mice (0.51% to 0.25%) (Fig. 5d), accompanied by the reduction of proliferating (S + G2/M) Igβ-Int cells (13.5% to 9.4%) (Fig. 5e).

Taken together along with the *in vitro* data, these results suggest that IL-21 may act on B cells directly or indirectly to down-regulate the expression of Igβ in the GC, which may be required for the effective progression of the cell cycle in these cells and/or for differentiation to the proliferating population.

### Blocking of Igβ down-regulation ameliorated autoimmune phenotype in BXSB-Yaa mice.

**Figure 6:**
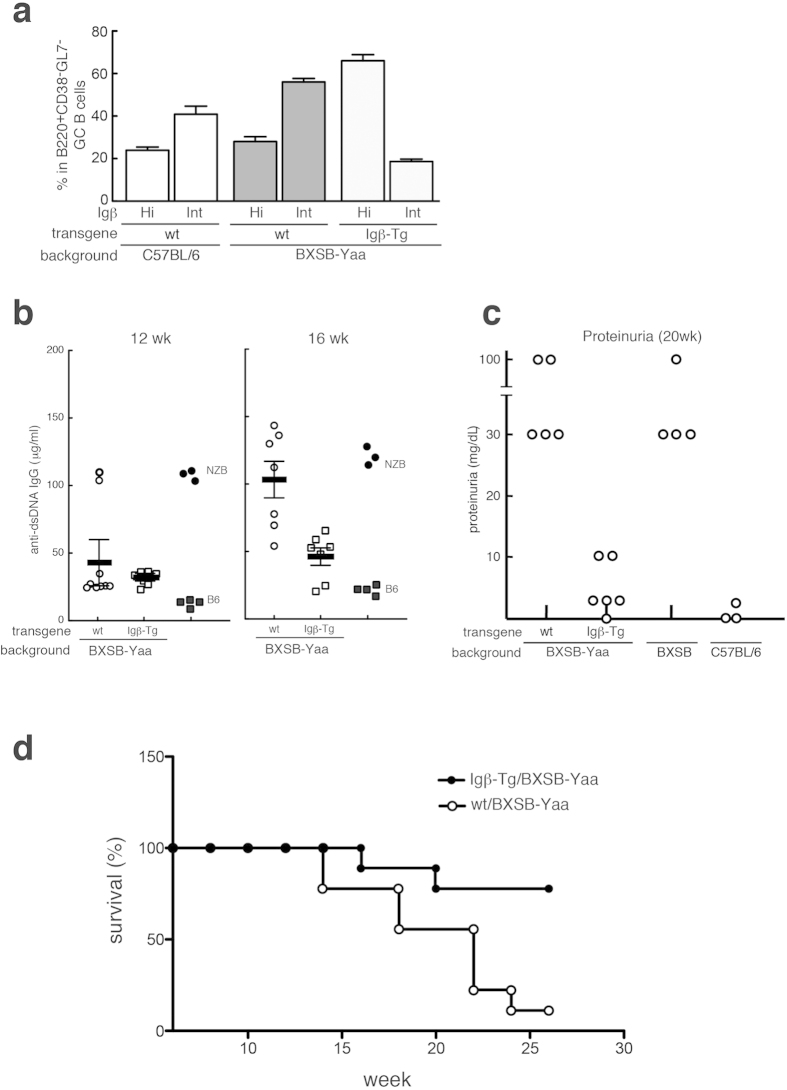
Amelioration of auto-reactive antibody production and proteinuria by suppression of Igβ down-regulation in BXSB-Yaa autoimmune prone mice. Igβ-Tg mice were back-crossed to BXSB-Yaa for 9 generation and obtained male mice were subjected to further analyses. Serum titers in NZB and wild type C57BL/6 mice were also measured concomitantly as positive band negative controls, respectively. (**a**) Percentages of Igβ-Hi and Igβ-Int B cell population in B220^+^CD38^−^GL7^−^ GC B cell subset from immunized C57BL/6 (open bars), BXSB-Yaa background wt (dark shaded bars) and Igβ-Tg (light shaded bars) mice are shown. (**b**) Serum titers of anti-dsDNA antibodies were measured in BXSB-Yaa background wt and Igβ-Tg mice at indicated ages. Each symbol indicate the serum levels for individual mouse. Thick lines indicate the average values and thin lines indicate SEM values. (**c**) Protineuria was measured at 20 weeks of age. Each circle indicate the serum levels for individual mouse. (**d**) Survival curve of BXSB-Yaa background wt and Igβ-Tg mice.

It has been reported that IL-21 plays an essential role in the phenotypes of BXSB-Yaa autoimmune-prone mice^27^,^28^. However, the precise mechanism of how IL-21 is involved in the generation and accumulation of autoreactive B cells has not been fully elucidated. To examine whether IL-21-mediated Igβ down-regulation may contribute to the pathogenesis of BXSB-Yaa mice, Igβ-Tg mice were back-crossed to this strain. The percentage of Igβ-Int cells among the B220^+^CD38^−^GL7^−^ GC B cell population in BXSB-Yaa mice was higher than in the wild type mice (56% to 41%), presumably due to the higher serum IL-21 level in BXSB-Yaa mice (Fig. 6a)^27^,^28^. When Igβ-Tg mice were back-crossed to BXSB-Yaa, their percentage decreased from 56% to 18.6% and that of Igβ-Hi cells increased reciprocally indicating that the Igβ transgene is expressed as expected in the BXSB-Yaa background (Fig. 6a). As shown in Fig. 6b,c, serum levels of anti-dsDNA IgG and proteinuria were ameliorated in BXSB-Yaa mice carrying the Igβ transgene. In addition, these mice tend to survive for a longer period compared with littermate BXSB-Yaa mice (Fig. 6d). Assuming that autoantibody-producing cells are derived from GC B cells^29^, these results suggest that the modulation of Igβ expression level affects not only positive selection, but also the accumulation of autoreactive B cells in the GC at least in the BXSB-Yaa back ground.

## Discussion

In this study, we demonstrated quantitatively that the cell surface expression of Igβ was more prominently lowered as compared with that of Igα in the murine GC B cells and that the inhibition of Igβ down-regulation resulted in the impairment of T-dependent immune responses. These results indicate that the down-regulation of Igβ in GC B cells is essential for optimal antibody production and affinity maturation. Firthermore, crossing Igβ-Tg mice with BXSB-Yaa autoimmune-prone mice ameliorated the pathogenic phenotype.

Clonal selection of GC B cells with different antigen specificities and/or affinities that were raised by the somatic hypermutation resulted in affinity maturation^11^,^30^. During this selection stage, each B cell did not continue to retain BCRs with different specificities and/or affinities on its surface simultaneously, and could change their BCR specificity promptly to be selected^31^. This leads to the hypothesis that there may exist a machinery that excludes pre-existing BCRs with low affinity from the cell surface. Based on previous reports that the concomitant expression of Igα and Igβ is required for the surface expression of IgM^4^,^5^,^6^, it can be assumed that the down-regulation of Igβ may contribute to the prompt clearance of surface IgM BCRs, most of which remain unmutated, in B cells that in turn start expressing mutated IgG in turn. Supporting this assumption, we have recently found that membrane-attached IgG1 can be expressed on the cell surface of GP2-293 cells and Igβ down-regulated Ramos B cells, in contrast to IgM which could not be expressed on the surface of these cells^32^.

It has been reported that somatic hypermutation plays an essential roles in the generation of autoreactive antibody-producing B cells in some case of SLE^33^,^34^,^35^. However, it was also revealed in these studies that in addition to the pathogenic somatic hypermutation, failure in the selection stage was also required for the onset of the diseases. When Igβ-Tg mice were crossed with BXSB-Yaa mice, the pathogenesis observed in these mice was ameliorated. Although the precise molecular mechanism underlying this ameliorated phenotype remains to be elucidated, it may be possible that in the Igβ-Tg background, class switching of GC B cells may proceeds preferentially through the IgM^+^GL7^+^Igβ-Hi population as in the case of *in vitro* class switching where expression of Igβ is sustained at a comparable level as the naive B cells (Fig. S2). Under such condition, a skewed positive selection for the self-antigen or affinity maturation that may affect the autoimmune symptoms may be partly bypassed because down-regulation of Igβ seems to be required for these processes to proceed efficiently.

Although the physiological relevance of Igβ down-regulation is clear, it remains to be determined how the expression of Igβ is modulated. In this study, it was revealed that Igβ down-regulation in the GC B cells was modulated at least in part at the transcription level (Figs. 1c and 2d). Regarding the transcription control elements in the Igβ promoter, three silencing motifs have been reported^36^,^37^. Among them, the most effective A+T-rich upstream sequence has been reported to bind Oct-1 and Oct-2^37^. Although these molecules are fascinating candidates for responsible taranscription factors, the direct involvement of Oct-1 and/or Oct-2 in the regulatory mechanisms of Igβ down-regulation in GC B cells remains to be tested.

With regard to soluble factors that may modulate Igβ expression, it was also revealed that Igβ down-regulation is partly mediated by IL-21 (Fig. 5). In accordance with these results, IL-21R-deficent mice showed impairment in the development of GC B cells and in affinity maturation^25^,^26^,^38^,^39^,^40^. However, because the blockade of Igβ down-regulation was not complete in IL-21R-deficient mice, it is evident that IL-21-independent machinery also exists. Antigen receptors of GC B cells are believed to be cross-linked by the cognate antigen during positive selection, which may result in the internalization of the BCR with its associating molecules, including Igα and Igβ. Thus, in addition to transcriptional regulation, we cannot completely rule out the possibility that post-translational modulation such as internalization, circulation or degradation of Igβ in association with BCRs may also be involved in its down-regulation^15^,^16^,^17^,^41^,^42^. Indeed, existence of an as-yet-unknown mechanism for post-transcriptional modulation of Igβ expression, which differ from Igα, was suggested in human GC B cells^17^. The surface expression levels of Igα were not dramatically down-regulated in the B220^+^CD38^−^GL7^−^ GC B cell population as compared with that of the B220 ^+^ CD38 ^+^ GL7^−^ B cells, in contrast to the expression levels of Igβ. However, a population that was up-regulating the surface Igα level could be observed, suggesting that still-unknown, unique mechanism exists for the regulation of Igα. Despite the marginal down-regulation of Igα in GC B cells, a decrease in the Igα mRNA level in GC B cells was detected (data not shown). This also suggests the existence of post-transcriptional regulation in the case of Igα. However, as aforementioned, a simple explanation of our results mainly by endocytosis of the IgM BCR complex associating with Igα/Igβ heterodimer seems unlikely.

## Methods

### Mice and immunization

Igβ-Tg mouse was generated using pEMV4C8-Igβ, which consists of mouse Eμ enhancer, mouse VH4C8 promoter, rabbit β-globin intron followed by HA-tagged mouse Igβ cDNA and rabbit β-globin derived polyA signal. Expression vector was a generous gift from Dr. Takeshi Watanabe. Linearlized expression vector was microinjected to C57BL/6 blastocyst and 2 founder lines, which both possess 3 copies of transgene, were established (Fig. S3) (PhoenixBio). IL-21 receptor-deficient mice^24^ was provided from Drs. Warren J. Leonard and Katsutoshi Ozaki. Immunizations were carried out as described previously^43^. In brief, 8–12 weeks old C57BL/6 mice were injected i.p. with 100ug NP-CGG (Biosearch Technologies) adsorbed to 3 mg Imject Alum (Thermo Fisher Scientific) according to the manufacturer’s instructions. All experimental protocols were approved by animal research committee in the Graduate School of Medicine, Kyoto University and carried out in accordance with the guidelines for the animal experiments in Kyoto University.

### Flow cytometry and histological analysis

All cells were stained with indicated combinations of antibodies, and analyzed with FACSAria II (BD Biosciences). Cells were stained in combination with propidium iodide (Sigma) to exclude dead cells as described previously^43^.

Frozen 8 μm sections of spleens from immunized mice were subjected to immunohistochemical staining. In brief, sections were fixed with 1:1 mixture of methanol (Wako) and acetone (Wako), blocked with 1% BSA in PBS for 1 h, and stained with indicated antibodies in PBS with 0.1% BSA for 1 h. Finally, the sections were washed and mounted with SlowFade Gold (Invitrogen). Stained sections were visualized using LSM 710 NLO confocal fluorescent microscope with ZEN software (ZEISS).

### Quantitative RT-PCR and PCR for cloning

Total RNA from sorted splenic cells was extracted using TRIzol Reagent (Invitrogen). Then, cDNA was synthesized from total RNA using SuperScript III First-Strand Synthesis System (Invitrogen). Transcription levels of Igβ, Bcl-6, AID, germline transcript from Iμ locus (GLT-μ), germline transcript from Iγ1 locus (GLT-γ1) and postswitch transcript of Cγ1 were determined by quantitative RT-PCR using the LightCycler 480 SYBR Green I Master and LightCycler 480 system (Roche). Relative expression levels of mRNA were calculated compared to those of β-actin.

### ELISA

Serum anti-NP antibody levels were measured by ELISA as described below. 96-well flat-bottom plates (Thermo Fisher Scientific) were coated with 10 μg/ml NP_54_-BSA (Biosearch Technologies), and then the wells were blocked with 5-fold diluted Blocking-One (Nacalai Tesque). Appropriately diluted serum samples were added to the wells and incubated at room temperature for 2 h. The plates were washed and incubated for another 1 h with diluted anti-IgM, -IgG1, -IgG2c and -IgG2b antibodies conjugated with horse radish peroxidase (Bethyl Laboratories), respectively. Bound secondary antibody was assessed by incubation with ABTS substrate (Sigma-Aldrich). Absorbance at 405 nm (A405) in each well was measured.

For the analysis of affinity maturation, appropriately diluted serum samples were assayed using an NP_4_-BSA (Biosearch Technologys) -coated 96-well plate and an NP_54_-BSA-coated plate. The A405(NP_4_)/A405(NP_54_) ratio was calculated as relative affinity.

### Generation of polyclonal antibody to cell surface Igα

Synthetic peptide corresponding to 89 to 99 amino acids with a extra cystein residue at N-terminal (CPEVNKNHRGLY) was conjugated to KLH and repeatedly immunized to the rabbits and the obtained anti-serum was affinity purified with the immunized peptide conjugated to BSA (Scrum). Specificity of the antibody was confirmed by western blot analysis of murine splenic B cells and surface staining of NIH/3T3 cells transfected with Igα, Igβ and single chain EGFP-Fcγ1. Also it was confirmed that no other spleen cells except B220^+^ cells were stained with the antibody.

### Analysis of somatic hypermutation

Total RNA from the sorted splenocytes of immunized C57BL/6 or Igβ-Tg mice was extracted and reverse transcribed to obtain cDNA, which was subjected to PCR using KOD DNA polymerase (Toyobo) with VH186.2-specific VH186.2F and IgG1-specific IgG1R primers and cloned into the plasmid PCR-blunt TOPO (Invitrogen) to analyze the sequence of the VH gene.

### Measurement of autoantibody and proteinuria

Serum anti-dsDNA titers were measured using mouse anti-dsDNA IgG ELISA kit (Alpha Diagnostic International Inc.) according to the manufacturer’s instruction. Proteinuria was measured using Albustix Reagent Strips (SIEMENS) according to the manufacturer’s instruction.

### Antibodies and oligo nucleotides

Antibodies and oligo nucleotides used in this study were listed in Table 1 and Table 2, respectively.

## Additional Information

**How to cite this article**: Todo, K. *et al*. Modulation of Igß is essential for the B cell selection in germinal center. *Sci. Rep.* **5**, 10303; doi: 10.1038/srep10303 (2015).

## Supplementary Material

Supplementary Information

## Figures and Tables

**Table 1:** List of antibodies used in the experiments

Antibody	**Manufacturer**	**clone**
anti-B220-APC-Cy7	BD Biosciences	RA3-6B2
anti-CD38-PE	eBioscience	90
anti-GL7-Alexa Fluor 647	eBioscience	GL-7
anti-CD79b-FITC	BD Biosciences	HM79b
anti-Fas-PE-Cy7	eBioscience	Jo2
anti-IgM-PE-Cy7	eBioscience	Il/41
streptavidin-PE-Cy7	eBioscience	-
streptavidin-Texas Red	BD Biosciences	-
anti-IgG1	BD Biosciences	A85-1
anti-IgG1-Pacific Blue	BD Biosciences	A85-1
anti-CD79b-biotin	BD Biosciences	HM79b
PNA-FITC	Seikagaku Kogyo	-

**Table 2:** List of the oligos used in the experiments

**Name of the oligo**	**Sequence of the oligo**
Igβ-qPCRF	CGAGGTTTGCAGCCAAAAAG
Igβ-qPCRR	CACAATGCGTCCCTCTTCTG
Bcl-6-qPCRF	ATCTGTGGCACTCGCTTCC
Bcl-6-qPCRR	AGTCGCAGTTGGCTTTTGTG
AID-qPCRF	AAGCCTGGGAAGGGCTACA
AID-qPCRR	GACATTCCAGGAGGTTGCTTTC
GLTμ-qPCRF	CTCTGGCCCTGCTTATTGTTG
GLTμ-qPCRR	GAAGACATTTGGGAAGGACTGACT
GLTγ1-qPCRF	AGGAATGTGTTTGGCATGGAC
GLTγ1-qPCRR	CACTGTCACTGGCTCAGGGAA
postswitch Cγ1-qPCRF	CTCTGGCCCTGCTTATTGTTG
postswitch Cγ1-qPCRR	CACTGTCACTGGCTCAGGGAA
β-actin-qPCRF	GGCTGTATTCCCCTCCATCG
β-actin-qPCRR	CCAGTTGGTAACAATGCCATGT
VH186.2F	TTCTTGGCAGCAACAGCTACA
IgG1R	CACTGTCACTGGCTCAGGGAA
