# Supplementary material for: Modulation of Igβ is essential for the B cell selection in germinal center
Source: Sci Rep. 2015 May 18;5:10303. doi: 10.1038/srep10303 (PMC4650814; doi:10.1038/srep10303)
Supplement: Supplementary Information [file srep10303-s1.pdf]

Supplementary information

title :

Modulation of Igβ is essential for the B cell selection in germinal center.

Authors :

Kagefumi Todo, Orie Koga, Miwako Nishikawa and Masaki Hikida

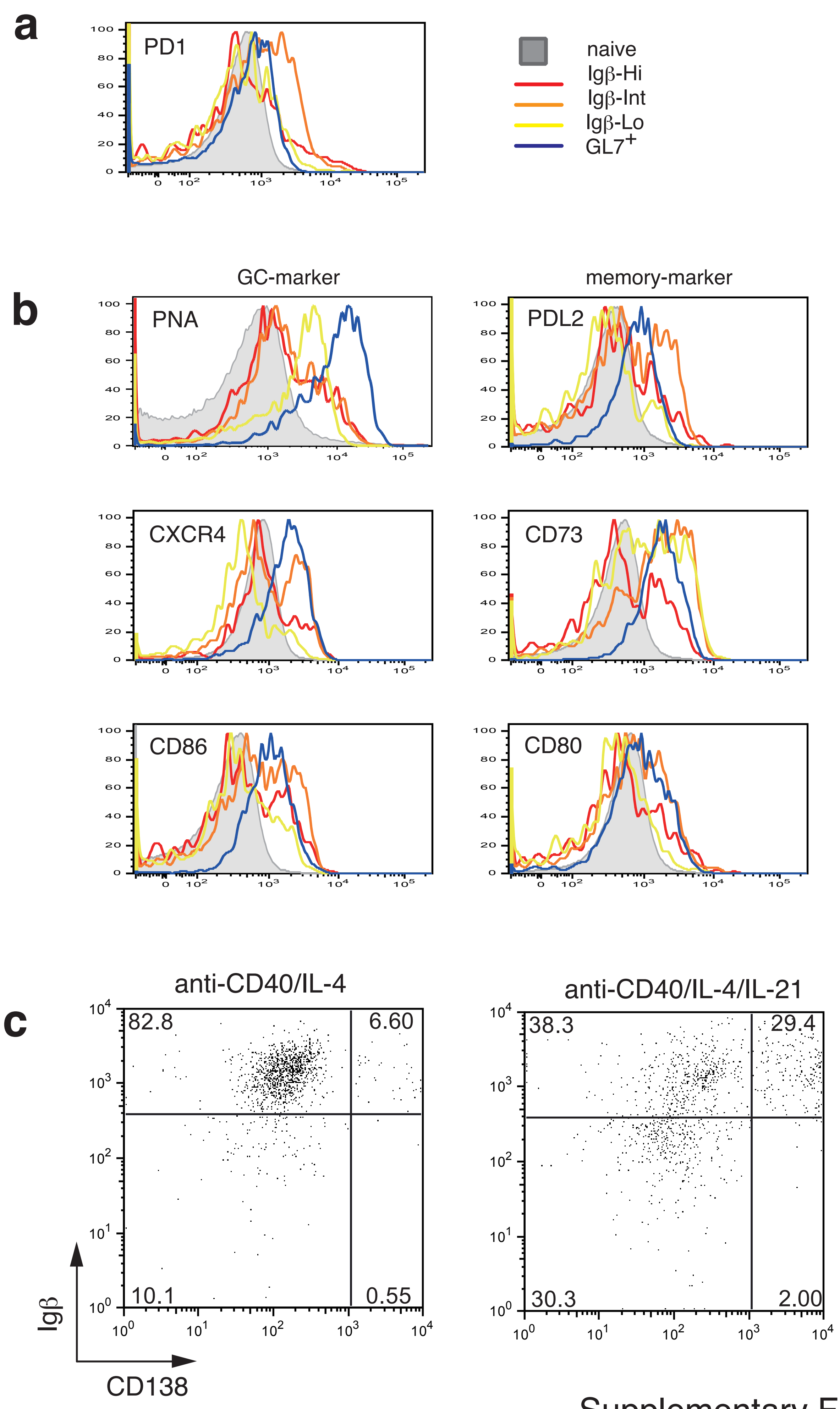

Supplementary Figure S1

Supplemetary Figure S1 Cell surface staining of B220^{+}CD38^{-}GL7^{-} population. B220^{+}CD38^{-}GL7^{-} and B220^{+}CD38^{-}GL7^{+} cells were stained with several GC- and memory-markers. (**a**) Staining pattern of PD1 (**b**) Staining patterns of PNA, CXCR4 and CD86 for the GC markers, and PDL2, CD73 and CD80 for the memory markers. (**c**) Defective expression of CD138 in Igβ down-regulated B cells. Splenic B cells were stimulated *in vitro* with anti-CD40 and IL-4 with or without IL21. After the culture, B cells were recovered and stained for Igβ and CD138. Cells were stained with propidium iodide to exclude the dead cells.

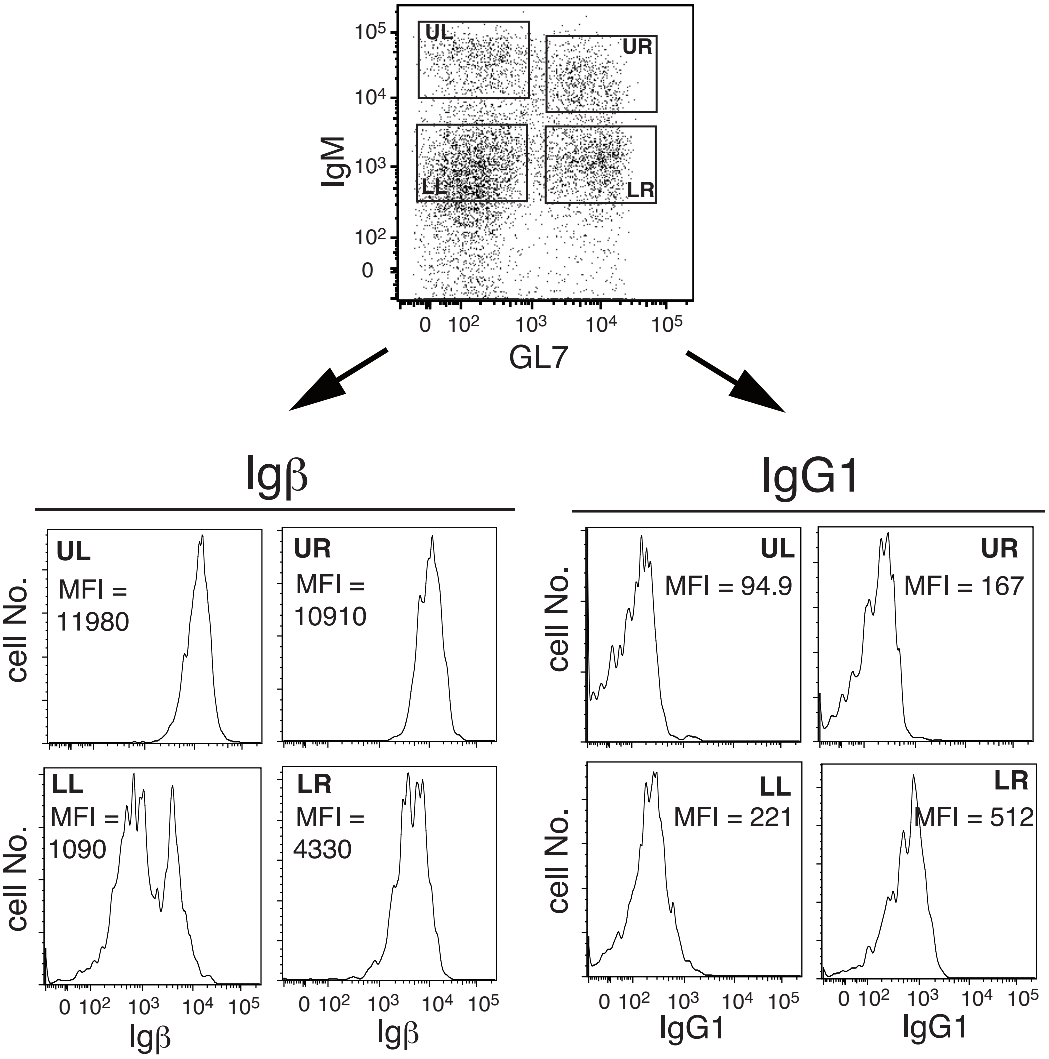

Supplementary Figure S2

Supplementary Figure S2 Possible two pathways of class switching in GC B cells. $B220^{+}CD38^{-}$ GC B cells were analyzed for their surface expression of IgM and GL7 (**top**). Expression levels of Igβ (**bottom left**) and IgG1 (**bottom right**) were examined in upper left (UL), upper right (UR), lower left (LL) and lower right (LR) gates indicated in the top panel. Mean fluorescence intensity (MFI) values in each panel are shown.

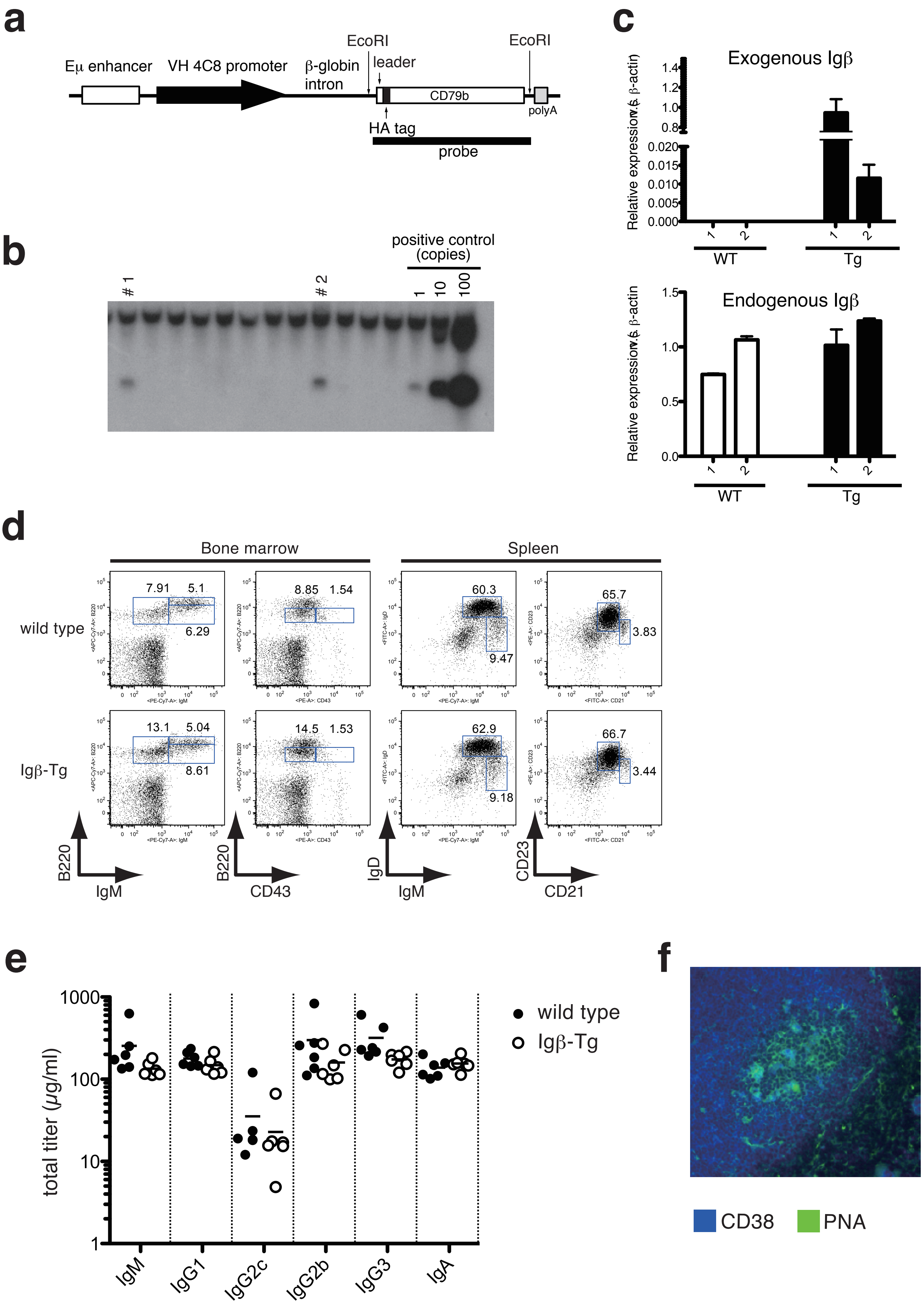

Supplementary Figure S3

Supplementary Figure S3 Generation of Igβ-Tg mice. (**a**) Schematic construct of Igβ expression cassette. Igβ cDNA was HA-tagged in order to distinguish the expression of introduced gene from the endogenous Igβ gene. (**b**) Southern blot analysis of genomic DNA from Igβ-Tg founder candidates. Tail DNA samples from each mouse were digested with EcoRI, electrophorased and blotted to the membrane. Then the membrane was blotted with the ^{32}P-labeled DNA probe fragment indicated in (**a**). (**c**) Expression levels of exogenous Igβ in Igβ-Tg mice. Total RNA was extracted from splenic B cells of unimmunized Igβ-Tg line (closed bar) and corresponding litter mates without transgene (open bar). Obtained RNA was subjected to quantitative RT-PCR. (**d**) Development of B cells in bone marrow and spleen was examined by flow cytometry with indicated cell surface markers. The numbers indicate the percentages of the cells in each square gate. (**e**) Basal Ig levels were analyzed by ELISA in unprimed Igβ-Tg (open circle) and wild type litter mates (closed circle). Filled and open circles indicate the value of each individual mouse. Bars indicate the mean values for each group. (**f**) Normal formation of GC in immunized Igβ-Tg mice. Frozen spleen sections were prepared at 10 day post immunization.

Sections were stained with anti-CD38 and PNA.
